# FAM83A as a Potential Biological Marker Is Regulated by miR-206 to Promote Cervical Cancer Progression Through PI3K/AKT/mTOR Pathway

**DOI:** 10.3389/fmed.2020.608441

**Published:** 2020-12-04

**Authors:** Li Rong, Haiyu Li, Zhaodong Li, Jing Ouyang, Yongping Ma, Fangzhou Song, Yaokai Chen

**Affiliations:** ^1^Chongqing Public Health Medical Center, Chongqing, China; ^2^Chongqing Medical University, Chongqing, China

**Keywords:** cervical cancer, FAM83A, prognosis, biomarker, miR-206, survival

## Abstract

**Background and Objective:** Chemotherapy and radiotherapy are effective treatment options for cervical cancer (CC), but their efficacy is limited by short survival rate of about 5 years particularly for advance stage CC. Bioinformatics analysis combined with experimental *in vivo* and *in vitro* data can identify potential markers of tumorigenesis and cancer progression to improve CC prognosis and survival rate of the patients. This study aims to investigate the prognostic value of family with sequence similarity 83, member A (FAM83A) gene and miR-206 in promoting CC progression and the involved genetic signaling pathways.

**Method:** This was a bioinformatic analysis study based on RNA sequencing data of The Cancer Genome Atlas (TCGA) and Gene Expression Omnibus (GEO) databases and verification by *in vivo* and *in vitro* experimental data. It was designed to evaluate whether the aberrantly expressed gene signatures could serve as new potential biomarker to improve prognosis prediction in CC. The TCGA RNA sequencing data [306 cervical squamous cell carcinoma (SCC) and endocervical adenocarcinoma samples and 13 adjacent samples] and GEO data (GSE9750 and GSE52903 datasets) were integrated and performed a bioinformatics analysis.

**Results:** The results showed that CC-associated FAM83A gene serves as a key regulator of CC development and progression. Functionally, we observed that FAM83A is significantly overexpressed in CC, which is linked to poor overall survival as well as disease-free survival in CC patients. The *in-vitro* and *in-vivo* assessments performed after silencing FAM83A revealed that cell proliferation was significantly inhibited and the S-phase cell cycle arrest was induced. Mechanistically, FAM83A plays a role in PI3K/AKT signaling, and its downstream molecules could promote CC cell proliferation. Furthermore, functionality assessments by *in-vitro* luciferase reporter system and immunoblot analysis showed that miR-206 was the upstream of FAM83A and negatively correlated with FAM83A.

**Conclusion:** The miR-206/FAM83A/PI3K/AKT signaling pathway possibly serves as a critical effector in CC progression indicating the potential prognostic value of FAM83A gene as a novel biomarker for CC progression.

## Background

Cervical cancer (CC) is the fourth most common female cancer and the second most prevalent cancer among young women (age group: 15–44 years old) worldwide accounting for approximately 570,000 new cases each year (1–5). Although great efforts on human papilloma virus (HPV) vaccines have been made to protect women from CC, it is still the second leading cause of tumors specific to women, next only to breast cancer (6–8). Developing countries account for 80% of the world's breast cancer cases. Squamous cell carcinoma (SCC) is the most prevalent histological type of CC accounts for approximately 80% of all CCs. Despite the relatively high mortality of CCs, little is known on definitive diagnosis and prognosis markers of these cancers (9).

Prognostic and diagnostic biomarkers play crucial roles in predicting the treatment response, prognosis and disease progression in cancer, developing new therapies, and elucidating tumorigenesis mechanisms (10, 11). High throughput profiling methods including next-generation sequencing and gene microarray have shown great potentials for identifying reliable prognostic biomarkers for different cancers (12, 13). The use of bioinformatics for analysis of gene sequencing and expression data can help developing prognostic and diagnostic biomarkers for cancers including CCs. Genotype-Tissue Expression (GTEx) and The Cancer Genome Atlas (TCGA) projects consisting of excellent databases of a very large RNA sequence data of cancerous and normal samples, provide good opportunities for high throughput modeling and bioinformatics analysis to determine diagnostic and prognostic biomarkers of cancer (9, 14, 15). TCGA database covers changes in 33 key cancer genomes and contains more than two petabytes of genomic data that are publicly available to help improve cancer prevention, diagnosis, and treatment (16–21). Different bioinformatics studies using the datasets of TCGA and Gene Expression Omnibus (GEO) gene microarrays have analyzed the gene sequencing and expression profiles of different tumors and demonstrated that abnormal overexpression or genes are key factors involved in the cancer progression (22, 23).

Family with sequence similarity 83, member A (FAM83A) that was first identified as a potential tumor-specific gene a bioinformatics approach is located on chromosome 8q24 (24). Previous studies have shown that FAM83A is aberrantly expressed in several human cancers including pancreatic, lung, breast, testis and bladder cancers (23, 25–29) indicating that FAM83A could possibly play an oncogenic role during the development and progression cancer. It has been shown that FAM83A is significantly overexpressed and associated with poorer overall survival (OS) and disease-free survival (DFS) in specific cancers including lung, breast, and pancreatic cancer. For instance, overexpression of FAM83A markedly facilitated, whereas inhibition of FAM83A decreased, cancer stem cell (CSC)-like features and chemoresistance both *in vitro* and in an *in vivo* mouse model of pancreatic cancer (23).

Different high-throughput modeling studies have analyzed the gene sequencing and expression profiles of CC and reported that aberrantly expressed genes are key factors involved in the cancer progression (22, 23). However, reviewing the literature shows that the differentially expressed genes (DEGs) associated with CC were rarely reported. In this regard, TCGA sequencing and GEO gene microarrays along with bioinformatics analysis can be employed to identify the DEGs affecting the biological functions of CC at the genetic level.

In the present study, we integrated the TCGA RNA sequencing data (306 cervical SCC and endocervical adenocarcinoma samples and 13 adjacent samples) and GEO data comprising the GSE9750 and GSE52903 datasets and performed a bioinformatics analysis. We identified the FAM83A gene is closely related to CC and further studied its biological function on CC and potential molecular regulatory mechanism.

## Materials and Methods

### RNA Sequencing and Microarray Data Analysis

The cervical SCC data and GTEx RNA sequencing data and a CC gene expression microarray comprising the GSE9750 and GSE52903 datasets were downloaded from the TCGA (https://cancergenome.nih.gov/abouttcga) and GEO (https://www.ncbi.nlm.nih.gov/geo/) datasets, respectively. The Limma package of R/bioconductor (bioconductor, USA) was used to screen the DEGs (settings: *q* < 0.05, |log2(fold change)|≥4). Hierarchical clustering and visualization were performed by the heatmap package of R.

### Immunohistochemistry Analysis

The surgical specimens examined in this study included 31 cervical SCC tissues and 31 corresponding para-carcinoma tissues obtained from the First Affiliated Hospital of Changde Vocational and Technical College between July 2013 and June 2015. Immunohistochemistry (IHC) analysis was carried out by initially dewaxing and rehydrating slides. This was followed by subjection to heat-induced epitope retrieval in citrate buffer. Incubation of slides with a rabbit anti-FAM83A polyclonal antibody (bs-16014R, BIOSS) at 4°C overnight was then carried out. This was followed by staining of sections with DAB (Maixin Bio, China) for 5 min. The specific FAM83A *in situ* hybridization (ISH) signal was judged as brown spots, and Image-Pro Plus 6.0 software was used to evaluate the expression level (30, 31).

### Cell Culture

Human cervical epithelial cells (CerEpiC), Human CC cell lines HeLa, SiHa, and CaSki (purchased from the Shanghai cell bank, Chinese Academy of Sciences) were cultured in Dulbecco's Modified Eagle Medium (HyClone, USA) containing 10% fetal bovine serum (HyClone, USA), 100 U/mL penicillin, 100 mg/mL streptomycin at 37°C in an environment containing 5% CO_2_.

### RNA Interference

The sequences of the small interfering RNAs of FAM83A (GenePharma, China), control siRNA sequences (GenePharma, China), short hairpin RNA (GenePharma, China) were listed, respectively as follows:

Sense: 5′-GGGCUGACUUUAGUGACAA-3′

Antisense: 5′-UUGUCACUAAAGUCAGCCC-3′

Sense: 5′-UUCUCCGAACGUGUCACGUTT-3′

Antisense: 5′-ACGUGACACGUUCGGAGAATT-3′

Sense: 5′-CACCGGGCTGACTTTAGTGACAACGAATTGTCACTAAAGTCAGCCC-3′

Antisense: 5′-AAAAGGGCTGACTTTAGTGACAATTCGTTGTCACTAAAGTCAGCCC-3′

The siRNA underwent transfection using Lipofectamine RNAiMAX Reagent (Invitrogen) according to the instructions. The Lentviral3-GFP-shRNA was used for infection of CaSki cells for 24 h, then culturing of cells in DMEN medium containing puromycin (3 μg/mL) was carried out, in order to form a stable cell line knocking down FAM83A.

### *In vitro* Cell Proliferation and Cell Cycle Assays

Cell activity was examined by CCK-8 Kits (Dojindo Laboratories, Japan) following transient transfection of siRNA for 24, 48, and 72 h. Treated cells were collected and underwent washing with phosphate-buffered saline. This was followed by fixation with 70% ethanol for 30 min. Next, it was incubated with 100 μL of RNase A for 30 min, followed by staining with propidium iodide (PI) for a duration of 30 min in the dark. Then, cycle distribution was analyzed by flow cytometry (FCM; BD, Influx).

### *In vivo* Tumor Growth Assay

All procedures of this study were approved by local ethics committee “Animal Care and Use Committee” of Chongqing Medical University, Chongqing, China which were in complete accordance with the regulations of “Guide for the Care and Use of Laboratory Animals.” The BALB/c female nude mice (*n* = 6), 4–6 weeks of age, were obtained from the “Experimental Animal Center” of Chongqing Medical University, Chongqing, China. The nude mice were randomized into control and experimental groups. In total, subcutaneous injection of 1 × 10^6^ shCtrl and shFAM83A CaSki cells were administered into the back of nude mice. Tumor growth was observed weekly for 5 weeks. This was followed by measurement and calculation of the tumor volume. After 5 weeks, mice were sacrificed, then tumors were excised and weighed. All of the data were represented as mean ± standard deviation (SD).

### Luciferase Reporter Assay

With reference to our previous experimental methods (32), synthesis of the wild-type and mutant fragment sequences of the 3'-untranslated region (UTR) of FAM83A were carried out followed by cloning into the pmirGLO vector (Promega, Madison). Cells inoculation in 24-well plates was carried out followed by co-transfection with pmirGLO-FAM83A-wild-type (WT), pmirGLO-FAM83A-mut, and miR-206 mimics or a control by Lipofectamine 2000. After a period of 48 h, luciferase assays were done with a Dual Luciferase Reporter Assay System (Promega). Examination of Luciferase activity was then determined with the Dual Luciferase Assay Kit (Promega) as per protocol.

### Statistical Analysis

The data were presented as Mean ± SD for all statistical analyses and data presentation unless otherwise is expressed. Student's *t*-test was used to investigate the differences between the two groups; the association between FAM83A expression and clinicopathological factors was investigated by Chi-square test. *P* < 0.05 was taken as statistically significant difference.

## Results

### Identification of FAM83A as a Cervical Cancer-Specific Gene

**Figure 1:**
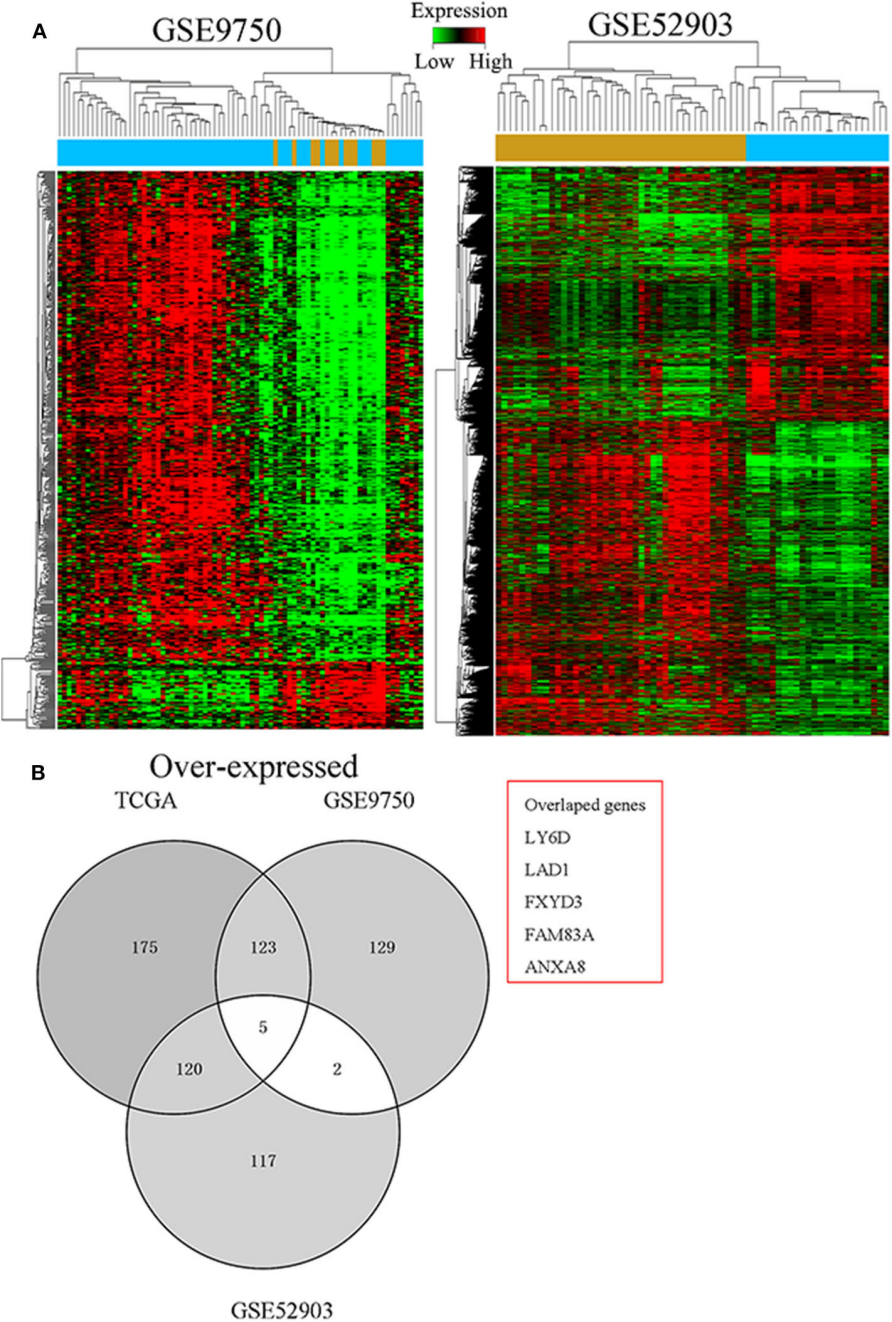
Identification of FAM83A as a cervical cancer-specific gene. **(A)** Hierarchical clustering analysis of genes that were differentially expressed (fold change > 4; *q* < 0.05) in cervical cancer and normal tissues. **(B)** Overlap of aberrantly expressed genes in the TCGA CESC data and GEO datasets.

To determine critical genes involved in the development of CC, the TCGA cervical SCC and GTEx RNA sequencing data and a CC gene expression microarray comprising the GSE9750 and GSE52903 datasets were analyzed. As seen in Figure 1A, we identified 252 misregulated genes in the TCGA database, 144 in the GSE9750 dataset, and 170 in the GSE52903 dataset (fold change > 4.0, *q* < 0.05). It was then discovered that, in all datasets, five genes were consistently overexpressed and three genes consistently under-expressed (Figure 1B). In the preliminary experiments, we detected the expression of these five up-regulated genes in CC tissues and cells by PCR, and found that FAM83A was most consistent and significant both in tissues and cells. Therefore, FAM83A was selected for further experimental validation and analyzed its biological behaviors in CC.

### FAM83A Is Overexpressed in Cervical Cancer and Correlates With Patient Survival

**Figure 2:**
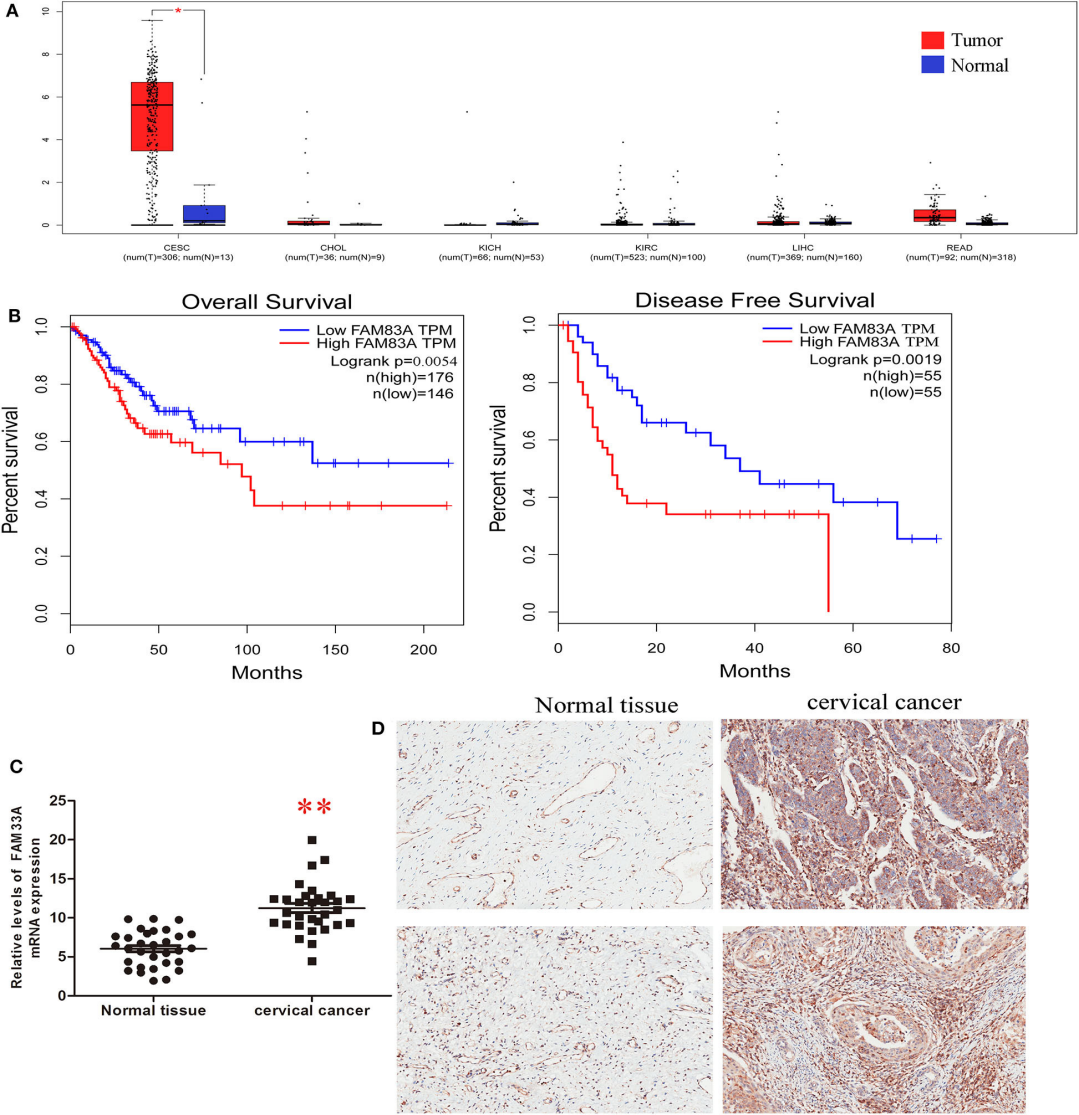
FAM83A is overexpressed in cervical cancer. **(A)** Analyses of FAM83A expression levels in cervical SCC and endocervical adenocarcinoma (CESC), cholangiocarcinoma (CHOL), kidney chromophobe (KICH), kidney renal clear cell carcinoma (KIRC), liver hepatocellular carcinoma (LIHC), and rectal adenocarcinoma (READ) samples using TCGA sequencing data. **(B)** Kaplan–Meier analysis of overall survival and disease-free survival in patients with cervical cancer (*P* < 0.001 for both overall survival and disease-free survival) based on FAM83A expression. **(C)** qRT-PCR was performed to detect FAM83A expression in cervical cancer and matched normal tissues (*n* = 31). **(D)** IHC was performed to detect FAM83A expression in cervical cancer and matched normal tissues (*n* = 31).

We explored the potential role of FAM83A in CC tumorigenesis using different analyses. We analyzed the expression of FAM83A in six cancers (cervical SCC and endocervical adenocarcinoma, cholangiocarcinoma, kidney chromophore, kidney renal clear cell carcinoma, rectal adenocarcinoma, and liver hepatocellular carcinoma) using TCGA sequencing datasets. The result showed that FAM83A is upregulated in CC tissues (Figure 2A). Low FAM83A expression was predictive of better OS (log-rank *P* = 0.05) and better DFS (log-rank *P* = 0.0004) (Figure 2B). To further verify the expression of FAM83A in CC, then we used qRT-PCR and IHC assays for examination of the level of FAM83A in CC tissues and adjacent ones. It was found that FAM83A was highly upregulated in 80.6% (25/31) of CC tissues compared to adjacent tissues (Figures 2C,D). To study the relation between FAM83A and CC clinical characteristics, the patients were split in two groups based on FAM83A levels. To do so, immunohistochemical staining was used to statistically score the expression level of FAM83A in CC tissues, which was higher than the mean as the high expression group and lower than the mean as the low expression group. Statistical results showed no correlation between FAM83A expression and age, number of lymph nodes, size of tumors or clinical stage (*P* > 0.05). On the other hand, FAM83A expression was significantly correlated with the histopathological type (*P* < 0.05) as well as lymph node status (*P* < 0.05) (Table 1).

**Table 1:** Relationship between FAM83A expression and clinicopathological factors.

**Characteristic**	**FAM83A**		***P*-value**
	**High expression**	**Low expression**	
Age (year)			0.235
≥50	10	4	
<50	15	2	
Lymph node number			0.219
≥25	6	3	
<25	19	3	
Lymph node status			0.007
N0	20	1	
N1	5	5	
Histological type			0.0001
Malignant	25	6	
Normal	7	24	
Tumor size (cm)			0.646
<3	9	2	
≥3	16	4	
Clinical stage			0.146
I	4	2	
II	15	3	
III	6	1	

### FAM83A Inhibits Cell Viability and Induces Cell Cycle Arrest

**Figure 3:**
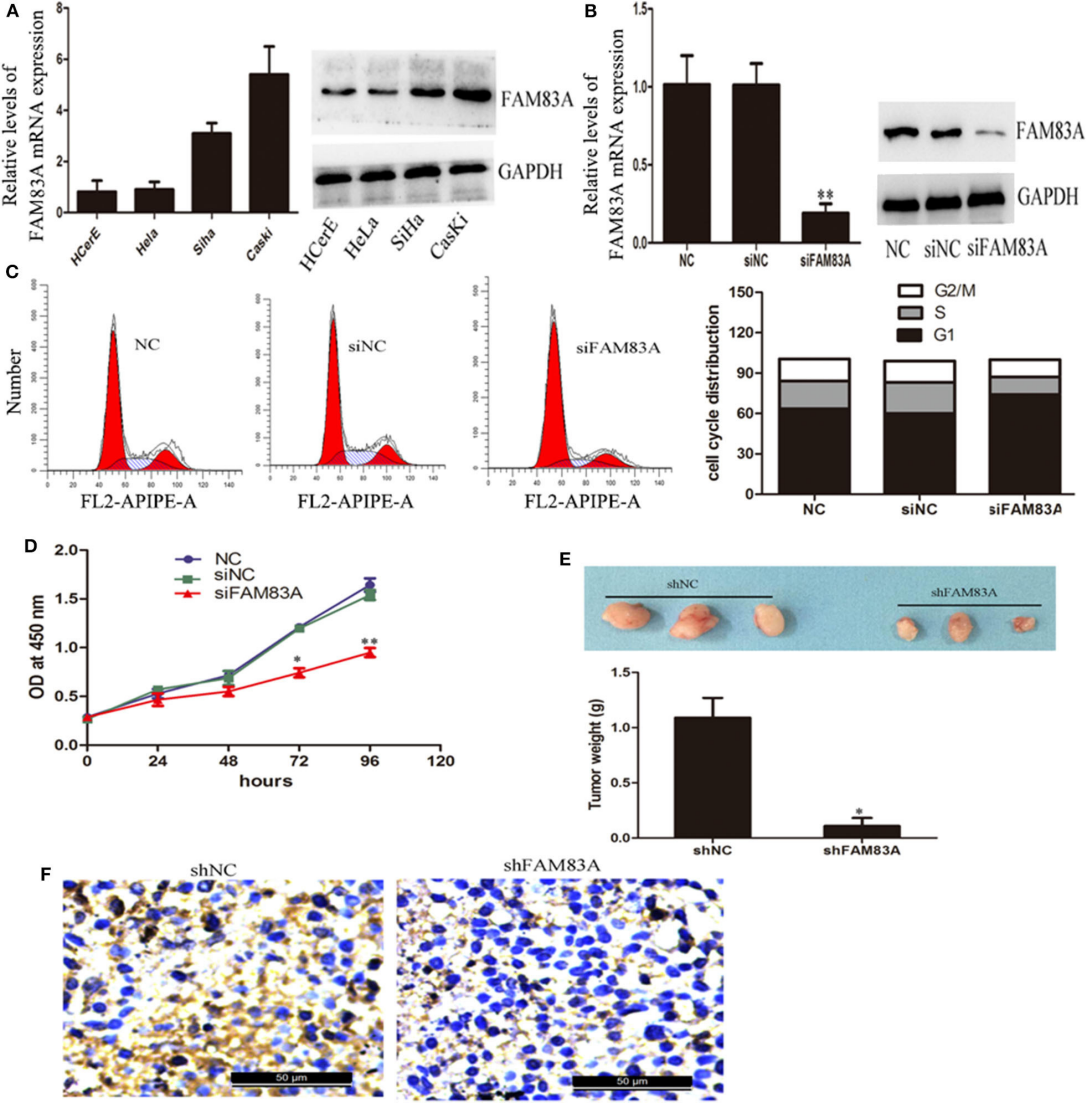
Knockdown of FAM83A expression inhibits cervical cancer cell proliferation *in vitro* and *in vivo*. **(A)** qRT-PCR and western blot analysis were performed to detect the expression of FAM83A in cervical cancer cell lines, normal human cervical epithelial cells (HCerEpiC), two low metastatic cervical cancer cell lines (HeLa and SiHa) and one highly metastatic cell line (CaSki). **(B)** qRT-PCR and immunoblot analysis confirmed that the FAM83A expression levels were significantly knocked down in CaSki cells. **(C)** FCM analysis of the effect of FAM83A knockdown on cell cycle distribution. **(D)** A CCK-8 assay was performed to detect cell proliferation of the control or siFAM83A-transfected CaSki cells. **(E)** Knockdown of FAM83A inhibits CaSki cell tumor growth *in vivo*. **(F)** Ki67 immunostaining of tumor samples from the FAM83A knockdown and control CaSki groups. Error bars represent the means of three independent experiments. ^*^P < 0.05, ^**^P < 0.01.

We used qRT-PCR and immunoblot analysis to detect the expression of FAM83A in HCerEpiC, highly metastatic cells (CaSki), as well as low metastatic cells (HeLa and SiHa). It was found that FAM83A expression was more in CC cell lines when compared to cervical epithelial cells. The CaSKi cells (high metastatic) the expression was the highest (Figure 3A). To further understand the biological role of FAM83A in regulation of CC cells, FAM83A was knocked down in CaSki cells (with high FAM83A expression) by siRNA/shRNA-mediated silencing. The qRT-PCR and immunoblot analysis confirmed that FAM83A expression levels were markedly reduced in CaSki cells (Figure 3B). As shown in Figure 3C, FCM was performed to determine whether FAM83A plays a role in regulating the cell cycle. FCM showed that the knockdown of FAM83A in CaSki cells resulted in a remarkable rise in the percentage of cells in G1 phase while reduced the proportion of cells in S-phase. These above results indicated that silence of FAM83A blocked the cell cycle from progressing and inhibited CC cells activity. Moreover, CCK-8 assays demonstrated that silencing FAM83A markedly inhibited the proliferation of CC cells *in vitro* (Figure 3D). For further confirmation of FAM83A role in the tumorigenesis of CC, nude mice were injected with FAM83A stable knockdown CaSki cells as well as control cells. FAM83A knockdown significantly restrained tumor growth in nude mice (Figure 3E). The IHC analysis showed that the FAM83A knockdown group had less Ki67-positive cells when compared with those from the control group (Figure 3F).

### PI3K/Akt Signaling Is Essential for FAM83A-Promoted CC Proliferation

**Figure 4:**
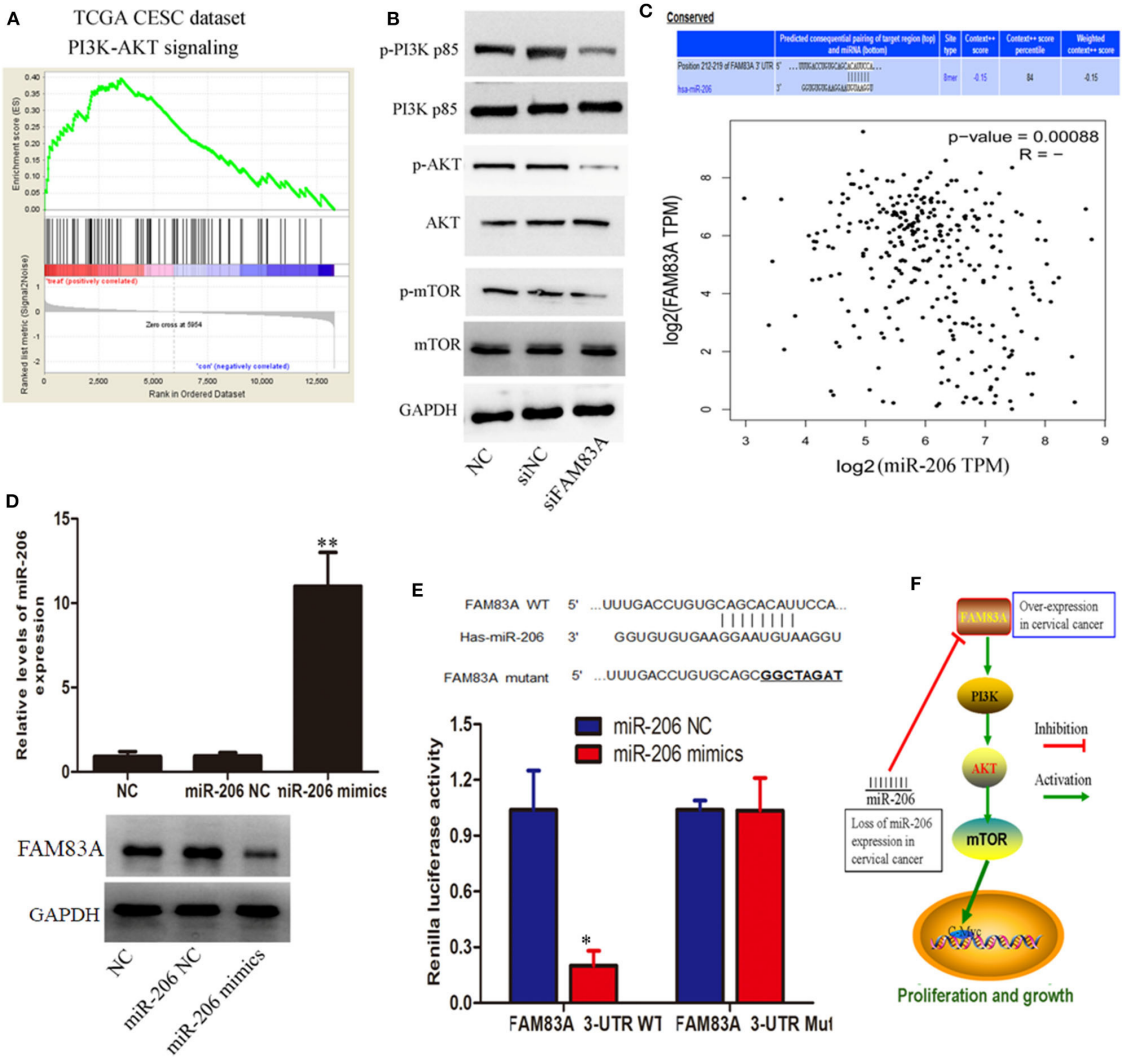
miR-206/FAM83A/PI3K/AKT signaling pathway molecules serve as critical effectors in cervical cancer progression. **(A)** GSEA plot showing that FAM83A expression was correlated with PI3K/AKT signaling-related gene signatures. **(B)** Protein levels of PI3K, p-PI3K, AKT, p-AKT, mTOR, and p-mTOR in CaSki cells are shown, and glyceraldehyde 3-phosphate dehydrogenase (GAPDH) was used as a loading control. **(C)** Schematic of binding sites in the FAM83A 3'-UTR complementary to the “seed region” of miR-206. Correlation analysis of FAM83A expression and miR-206. **(D)** The overexpression of miR-206 dramatically decreased the protein expression of FAM83A in CaSki cells. **(E)** The complementary sequences or the mutant (underlined) binding site of FAM83A or the miR-206 binding site in the 3'-UTR of FAM83A from different mammalian species. **(F)** Summary of the mechanism of FAM83A in cervical cancer. Error bars represent the means of three independent experiments. ^*^P < 0.05, ^**^P < 0.01.

For further elucidation of molecular mechanisms of proliferation inhibition as well as cell cycle arrest by FAM83A depletion in CC, a gene set enrichment analysis (GSEA) of publicly available TCGA cervical SCC data was performed. We were excited to find that FAM83A expression was correlated with the activation of phosphatidylinositol 3 kinase (PI3K)/protein kinase B (AKT) signaling (Figure 4A). Therefore, we hypothesized that FAM83A affects the activity of PI3K/Akt/mTOR signal path in CC cells. Subsequently, our western blot analysis demonstrated that FAM83A knockdown lead to significant dephosphorylation of PI3K, Akt, and mTOR in CaSki cells (Figure 4B).

### Loss of miR-206 Expression Induces FAM83A Overexpression in CC

To identify whether microRNAs (miRNAs) are involved in regulation of FAM83A in CC cells, we performed a bioinformatics analysis using the PicTar, miRanda (miRBase), and TargetScan in order to predict the potential microRNA regulating FAM83A. The miR-206 was selected to target FAM83A utilizing these above programs. Correlation analysis of FAM83A expression and miR-206 revealed that FAM83A has a significant negative correlation with miR-206 in the TCGA dataset (Figure 4C). Immunoblot analysis indicated that the overexpression of miR-206 dramatically decreased FAM83A protein expression in CaSki cells (Figure 4D). Subsequently, we constructed luciferase reporters including the putative miR-206 binding sites, which contain WT or mutated miR-206 binding sites. Experimental data indicated that overexpression of miR-206 weakened the luciferase activity of the WT reporter vector without effecting the mutant reporter vector (Figure 4E). In a word, these above experiments consistently indicated that loss of miR-206 expression increases the expression of FAM83A, which promotes cell proliferation *via* the PI3K/AKT/mTOR pathway and imposes an additional posttranscriptional modulation (Figure 4F).

## Discussion

In this present study, TCGA RNA sequencing data including cervical SCC and endocervical adenocarcinoma samples and paracancer samples and GEO data comprising the GSE9750 and GSE52903 datasets were analyzed using bioinformatics analysis. Five genes (KRT17, FXYD3, KRT5, FAM83A, and CLDN4) were consistently overexpressed and three genes (CDKN2A, MCM5, and RFC4) were under-expressed in these above three datasets.

We found that FAM83A is specifically overexpressed in CC tissue but not in cholangiocarcinoma, kidney chromophobe, kidney renal clear cell carcinoma, rectal adenocarcinoma, or liver hepatocellular carcinoma. From TCGA database, we predicted that FAM83A expression in cancerous tissue and paracancer tissue in cervical SCC and endometrial adenocarcinoma (CESC) was significantly different. We did not mention the difference of FAM83A expression between cervical SCC and endometrial adenocarcinoma. At this time, we aimed to explore the role of FAM83A in CC cancer and its prognostic value.

Some studies have investigated the roles of FAM83A in PI3K/AKT signaling pathways in cancer. Lee et al. reported that in the breast cancer FAM83A possibly contributes in exerting resistance to tyrosine kinase inhibitors *via* activating epidermal growth factor receptor (EGFR)/PI3K/AKT signaling pathway *via* interacting with c-RAF and phosphatidylinositol 3 kinase p85. These findings indicate that FAM83A overexpression might result in chemoresistance (33, 34). Similarly, both *in vitro* and *in vivo* models silencing FAM83A significantly reduces proliferation, anchorage-independent growth and metastatic capacities of breast cancer cells.

PI3K/AKT signaling pathway is an intracellular signal transduction pathway that plays roles in proliferation, metabolism, cell survival and growth, and angiogenesis in response to extracellular signals. This signaling pathway is mediated through serine and/or threonine phosphorylation of different types of downstream compounds, of them PI3K and AKT are the main proteins. The effects of FAM83A on PI3K/AKT pathway have been investigated in few cancers (22, 35–37). Liu et al. analyzed TCGA database and reported that FAM83A is overexpressed in hepatocellular carcinoma (HCC) cells and plays a cancer-promoting and treatment-resistance role. Their functional bioinformatics analyses indicated that FAM83A promoted the PI3K/AKT signaling pathway, its downstream c-JUN protein, and epithelial-to-mesenchymal transition (EMT)-related protein levels, including downregulation of E-cadherin and upregulation of Vimentin and N-cadherin. They reported that c-JUN induced FAM83A expression through direct binding to its promoter region, which forms a positive-feedback loop for FAM83A/PI3K/AKT/c-JUN. Liu et al. concluded that FAM83A serves as a tumorigenesis of HCC and promotes migration, invasion and metastasis through triggering a FAM83A/PI3K/AKT/c-JUN positive-feedback loop (38).

Hu et al. examined the roles and possible mechanism of FAM83A in non-small cell lung cancer (NSCLC) progression through bioinformatics analysis of GEO and TCGA databases and RT-PCR and reported high FAM83A expression in NSCLC that was associated with the poor prognosis (35). *In vitro* model showed that silencing FAM83A by siRNA/shRNA markedly reduced cell proliferation, induced cell apoptosis, and inhibited cell motility. *In vivo* experiments showed that silencing FAM83A in A549 cells reduced subcutaneous tumor growth and lung metastasis as well as phosphorylation of ERK and PI3K/AKT/mTOR (35). Contrary, FAM83A overexpression promoted cell proliferation and metastasis invasion *in vitro* that was suppressed by PI3K inhibitor and ERK inhibitor respectively. Their findings demonstrated that FAM83A promotes oncogenesis of NSCLC partly through ERK and PI3K/AKT/mTOR pathways (35).

Based on the expression level of FAM83A, we divided the patients into high and low-risk groups based on the median risk score in the TCGA dataset as a cut-off value, and the median OS and DFS in the low-risk group was pronounced higher of the one in the high-risk group. PCR and IHC results confirmed the expression characteristic of FAM83A in CC clinical samples. It was seen that the expression quantity of FAM83A was markedly correlated with histopathological type and lymph node status. Furthermore, our results demonstrated that silencing FAM83A markedly inhibited CC cell proliferation both in *in-vitro* as well as *in-vivo* experiments, meanwhile induced cell cycle arrest *in vitro*. FAM83A is a probable protooncogene that regulates the EGF/EGFR signaling pathway (39, 40) and seems vital for activating RAS/MAPK signaling cascade under the stimulation of EGFR. This gene also activates signaling cascades independent of EGFR regulation.

We performed a GSEA analysis on the cervical SCC samples of the TCGA database and found a regulatory relationship between FAM83A and PI3K/AKT pathway. The experiments demonstrated that FAM83A alters the activity of PI3K/Akt/mTOR signaling in CC.

MiRNAs have attracted considerable and wide attention in the regulation of gene expression because of their important status in cellular differentiation and embryonic stem cell growth. We performed a bioinformatics analysis using three different types of prediction software including PicTar, TargetScan, and miRanda (miRBase). We found that miR-206 directly suppresses FAM83A expression in CC. Subsequently, our western blot analysis and luciferase reporter assay consistently indicated that loss of miR-206 expression upregulated the expression of FAM83A. One interesting question is that overexpression of FAM83A or inhibition of miR-206 in cell lines such as HCerEpiC is enough to drive the cancer progression? And how do PI3K or AKT inhibitors influence such effects? We hypothesized that overexpression of FAM83A or miR206 in normal HCerEpiC might affect normal cell physiological function, which may be a driver for malignant transformation of normal cells. If extrapolated from our results, the addition of PI3K or AKT inhibitors may partially reverse the previously assumed effect. However, conducting further well-designed studies investigating such hypotheses is recommended.

## Conclusion

In conclusion, our current experimental research reveal that molecules involved in the miR-206/FAM83A/PI3K/AKT signaling pathway serve as critical effectors in CC progression, meanwhile, FAM83A, as a potential protooncogene is closely related to the survival and prognosis of CC, may serve as potential therapeutic targets in CC.

## Data Availability Statement

The datasets presented in this study can be found in online repositories. The names of the repository/repositories and accession number(s) can be found in the article/Supplementary Material.

## Ethics Statement

All procedures were in agreement with the Guide for the Care and Use of Laboratory Animals and approval was obtained by the Animal Care and Use Committee of Chongqing Medical University. All procedures performed in our research were in accordance with the related ethical standards.

## Author's Note

LR and HL are postdoctoral researchers jointly trained by Chongqing Public Health Medical Center and Chongqing Medical University.

## Author Contributions

LR, HL, and FS: conceptualization. LR, YM, and ZL: methodology. YM and HL: software. LR, YC, and FS: validation, resources, and project administration. LR, HL, and YM: formal analysis. LR and HL: investigation. LR, HL, and ZL: data curation. LR: writing-original draft preparation. YM, YC, and FS: writing-review and editing. HL and JO: visualization. JO and YC: supervision. LR and YC: funding acquisition. All authors contributed to the article and approved the submitted version.

## Conflict of Interest

The authors declare that the research was conducted in the absence of any commercial or financial relationships that could be construed as a potential conflict of interest.

## References

[1] Fitzmaurice C Abate D Abbasi N Abbastabar H Abd-Allah F Abdel-Rahman O. Global, regional, and national cancer incidence, mortality, years of life lost, years lived with disability, and disability-adjusted life-years for 29 cancer groups, 1990 to 2017: a systematic analysis for the global burden of disease study. JAMA Oncol. (2019) 5:1749–68. 10.1001/jamaoncol.2019.2996 31560378 PMC6777271

[2] Chen D Juko-Pecirep I Hammer J Ivansson E Enroth S Gustavsson I Feuk L Magnusson PK McKay JD WE and GU. Genome-wide association study of susceptibility loci for cervical cancer. J Natl Cancer Inst. (2013) 105:624–33. 10.1093/jnci/djt051 23482656

[3] Wilbur DC. Practical issues related to uterine pathology: *in situ* and invasive cervical glandular lesions and their benign mimics: emphasis on cytology–histology correlation and interpretive pitfalls. Mod Pathol. (2016) 29(Suppl. 1):S1–11. 10.1038/modpathol.2015.138 26715169

[4] Berti FCB Pereira APL Cebinelli GCM Trugilo KP Brajão de Oliveira K. The role of interleukin 10 in human papilloma virus infection and progression to cervical carcinoma. Cytokine Growth Factor Rev. (2017) 34:1–13. 10.1016/j.cytogfr.2017.03.002 28365229

[5] Wieringa HW van der Zee AGJ de Vries EGE van Vugt MATM. Breaking the DNA damage response to improve cervical cancer treatment. Cancer Treat Rev. (2016) 42:30–40. 10.1016/j.ctrv.2015.11.008 26643553

[6] Fetcko K Gondim DD Bonnin JM Dey M. Cervical cancer metastasis to the brain: a case report and review of literature. Surg Neurol Int. (2017) 8:181. 10.4103/sni.sni_111_17 28868193 PMC5569407

[7] Thomas M Narayan N Pim D Tomaić V Massimi P Nagasaka K. Human papillomaviruses, cervical cancer and cell polarity. Oncogene. (2008) 27:7018–30. 10.1038/onc.2008.351 19029942

[8] Gadducci A Guerrieri ME Greco C. Tissue biomarkers as prognostic variables of cervical cancer. Crit Rev Oncol/Hematol. (2013) 86:104–29. 10.1016/j.critrevonc.2012.09.003 23031678

[9] Tomczak K Czerwińska P Wiznerowicz M The Cancer Genome Atlas (TCGA): an immeasurable source of knowledge. Contemp Oncol. (2015) 19:A68–77. 10.5114/wo.2014.47136 PMC4322527 25691825

[10] Zheng H Zhang G Zhang L Wang Q Li H Han Y. Comprehensive review of web servers and bioinformatics tools for cancer prognosis analysis. Front Oncol. (2020) 10:68. 10.3389/fonc.2020.00068 32117725 PMC7013087

[11] Lu C Yang M Luo F Wu FX Li M Pan Y. Prediction of lncRNA-disease associations based on inductive matrix completion. Bioinformatics. (2018) 34:3357–64. 10.1093/bioinformatics/bty327 29718113

[12] Falzone L Candido S Salemi R Basile MS Scalisi A McCubrey JA. Computational identification of microRNAs associated to both epithelial to mesenchymal transition and NGAL/MMP-9 pathways in bladder cancer. Oncotarget. (2016) 7:72758–66. 10.18632/oncotarget.11805 27602581 PMC5341942

[13] Lánczky A Nagy Á Bottai G Munkácsy G Szabó A Santarpia L. miRpower: a web-tool to validate survival-associated miRNAs utilizing expression data from 2178 breast cancer patients. Breast Cancer Res Treat. (2016) 160:439–46. 10.1007/s10549-016-4013-7 27744485

[14] Anaya J OncoLnc: linking TCGA survival data to mRNAs, miRNAs, and lncRNAs. Peer J Comput Sci. (2016) 4:e1780v1 10.7287/peerj.preprints.1780v1

[15] Barrett T Wilhite SE Ledoux P Evangelista C Kim IF Tomashevsky M. NCBI GEO: archive for functional genomics data sets—update. Nucleic Acids Res. (2013) 41(D1):D991–5. 10.1093/nar/gks1193 23193258 PMC3531084

[16] Neapolitan R Jiang X. Inferring aberrant signal transduction pathways in ovarian cancer from TCGA Data. Cancer Inform. (2014) 13s1(Suppl. 1):CIN.S13881. 10.4137/CIN.S13881 25392681 PMC4216062

[17] Koehorst JJ van Dam J Saccenti E Martins dos Santos VAP Suarez-Diez M Schaap PJ. SAPP: functional genome annotation and analysis through a semantic framework using FAIR principles Bioinformatics. (2017) 34:1401–3. 10.1093/bioinformatics/btx767 29186322 PMC5905645

[18] Mamatjan Y Agnihotri S Goldenberg A Tonge P Mansouri S Zadeh G. Molecular signatures for tumor classification: an analysis of The Cancer Genome Atlas data. J Mol Diagn. (2017) 19:881–91. 10.1016/j.jmoldx.2017.07.008 28867603

[19] Weisenberger DJ. Characterizing DNA methylation alterations from the cancer genome atlas. J Clin Invest. (2014) 124:17–23. 10.1172/JCI69740 24382385 PMC3871233

[20] Mei Y Tang Z Li Z Yang X. Repeatability and reproducibility of quantitative corneal shape analysis after orthokeratology treatment using image-pro plus software. J Ophthalmol. (2016) 2016:1732476. 10.1155/2016/1732476 27774312 PMC5059590

[21] Li S Xu F Li H Zhang J Zhong A Huang B. S100A8^+^ stroma cells predict a good prognosis and inhibit aggressiveness in colorectal carcinoma. Oncoimmunology. (2017) 6:e1260213. 10.1080/2162402X.2016.1260213 28197382 PMC5283617

[22] Bartel CA Jackson MW. HER2-positive breast cancer cells expressing elevated FAM83A are sensitive to FAM83A loss. PLoS ONE. (2017) 12(5). 10.1371/journal.pone.0176778 28463969 PMC5413028

[23] Chen S Huang J Liu Z Liang Q Zhang N Jin Y. FAM83A is amplified and promotes cancer stem cell-like traits and chemoresistance in pancreatic cancer. Oncogenesis. (2017) 6:e300. 10.1038/oncsis.2017.3 28287611 PMC5533946

[24] Li Y Dong X Yin Y Su Y Xu Q Zhang Y. BJ-TSA-9, a novel human tumor-specific gene, has potential as a biomarker of lung cancer. Neoplasia. (2005) 7:1073–80. 10.1593/neo.05406 16354590 PMC1501171

[25] Cipriano R Miskimen KLS Bryson BL Foy CR Bartel CA Jackson MW. Conserved oncogenic behavior of the FAM83 family regulates MAPK signaling in human cancer. Mol Cancer Res. (2014) 12:1156–65. 10.1158/1541-7786.MCR-13-0289 24736947 PMC4135001

[26] Zhang J Sun G Mei X. Elevated FAM83A expression predicts poorer clinical outcome in lung adenocarcinoma. Cancer Biomark. (2019) 26:367–73. 10.3233/CBM-190520 31594212 PMC12826422

[27] Liu L Ma C Xu Q Cheng L Xiao L Xu D. A rapid nested polymerase chain reaction method to detect circulating cancer cells in breast cancer patients using multiple marker genes. Oncol Lett. (2014) 7:2192–8. 10.3892/ol.2014.2048 24932314 PMC4049700

[28] Richtmann S Wilkens D Warth A Lasitschka F Winter H Christopoulos P. FAM83A and FAM83B as prognostic biomarkers and potential new therapeutic targets in NSCLC. Cancers (Basel). (2019) 11:652. 10.3390/cancers11050652 31083571 PMC6562954

[29] Snijders AM Lee SY Hang B Hao W Bissell MJ Mao JH. FAM83 family oncogenes are broadly involved in human cancers: an integrative multi-omics approach. Mol Oncol. (2017) 11:167–79. 10.1002/1878-0261.12016 28078827 PMC5527452

[30] Khenkhar M Uhlig PC David KA Bigley AL Sherry L Digital image analysis of automated mRNA in situ hybridization and immunohistochemistry to quantify HER3 expression in cancer tissues. J Clin Oncol. (2016) 34(Suppl. 15):e23172.

[31] Ahmad N Haider S Jagannathan S Anaissie E Driscoll JJ. MicroRNA theragnostics for the clinical management of multiple myeloma. Leukemia. (2014) 28:732–8. 10.1038/leu.2013.262 24714346

[32] Li H Jia Y Cheng J Liu G Song F. LncRNA NCK1-AS1 promotes proliferation and induces cell cycle progression by crosstalk NCK1-AS1/MIR-6857/CDK1 pathway. Cell Death Dis. (2018) 9:198. 10.1038/s41419-017-0249-3 29416014 PMC5833418

[33] Lee SY Meier R Furuta S Lenburg ME Kenny PA Xu R. FAM83A confers EGFR-TKI resistance in breast cancer cells and in mice. J Clin Invest. (2012) 122:3211–20. 10.1172/JCI60498 22886303 PMC3428077

[34] Grant S. FAM83A and FAM83B: Candidate oncogenes and TKI resistance mediators. J Clin Invest. (2012) 122:3048–51. 10.1172/JCI64412 22886299 PMC3428099

[35] Hu H Wang F Wang M Liu Y Wu H Chen X. FAM83A is amplified and promotes tumorigenicity in non-small cell lung cancer *via* ERK and PI3K/Akt/mTOR pathways. Int J Med Sci. (2020) 17:807–14. 10.7150/ijms.33992 32218702 PMC7085261

[36] Xu J Lu W. FAM83A exerts tumor-suppressive roles in cervical cancer by regulating integrins. Int J Oncol. (2020) 57:509–21. 10.3892/ijo.2020.5078 32626940 PMC7307588

[37] Zhou F Geng J Xu S Meng Q Chen K Liu F. FAM83A signaling induces epithelial-mesenchymal transition by the PI3K/AKT/Snail pathway in NSCLC. Aging (Albany NY). (2019) 11:6069–88. 10.18632/aging.102163 31444970 PMC6738414

[38] Liu C Peng X Li Y Liu S Hou R Zhang Y. Positive feedback loop of FAM83A/PI3K/AKT/c-Jun induces migration, invasion and metastasis in hepatocellular carcinoma. Biomed Pharmacother. (2020) 123:109780. 10.1016/j.biopha.2019.109780 31901550

[39] Rhyasen GW Starczynowski DT. Deregulation of microRNAs in myelodysplastic syndrome. Leukemia. (2012) 26:13–22. 10.1038/leu.2011.221 21852786

[40] Contreras J Rao DS. MicroRNAs in inflammation and immune responses. Leukemia. (2012) 26:404–13. 10.103-8/leu.2011.356 22182919

