# Supplementary material for: FAM83A as a Potential Biological Marker Is Regulated by miR-206 to Promote Cervical Cancer Progression Through PI3K/AKT/mTOR Pathway
Source: Front Med (Lausanne). 2020 Dec 4;7:608441. doi: 10.3389/fmed.2020.608441 (PMC7746878; doi:10.3389/fmed.2020.608441)
Supplement: Supplementary file 1 [file Data_Sheet_1.pdf]

Table S1. The univaraite and multivariate Cox regression model (TCGA_CESC n=307)

Variable	Univariate regression		Multivariate regression	
	P value	Hazard ratio (95%CI)	P value	Hazard ratio (95%CI)
FAM83A (High vs Low)	0.0054	1.53(1.25~2.26)	0.27514595	1.3194(0.802~2.1707)
Age (ref = <=46)				
>46	0.60566274	1.1282(0.7136~1.7838)	0.9723201	0.9904(0.5751~1.7056)
Grade (ref = G1)				
G2	0.65658365	1.3068(0.4018~4.25)	0.67424098	0.7675(0.2235~2.6355)
G3	0.81319394	1.1565(0.3463~3.8617)	0.83858782	0.8775(0.2496~3.0855)
G4	0.99538217	0(0~Inf)	0.99786129	0(0~Inf)
Unknown	0.22521132	2.2232(0.6113~8.0861)	0.83698011	0.8582(0.2~3.6831)
Stage (ref = I)				
II	0.47108372	0.7808(0.3984~1.5304)	0.39129268	0.6694(0.2674~1.6759)
III	0.41495257	1.3225(0.6754~2.5896)	0.38685312	0.6927(0.3016~1.5911)
IV	4.2531E-06	4.2391(2.2905~7.8456)	0.13155865	2.177(0.792~5.9841)
Unknown	0.99482526	0(0~Inf)	0.99663513	0(0~Inf)
pM (ref = M0)				
M1	0.02432016	3.4696(1.1751~10.2445)	0.84098603	1.1528(0.2874~4.6246)
Unknown	0.01496401	1.9262(1.136~3.266)	0.16463955	1.5714(0.8307~2.9726)
pN (ref = N0)				
N1	0.00173806	2.8514(1.48~5.4935)	0.01018997	2.5214(1.2452~5.1054)
Unknown	0.00017969	2.9878(1.6852~5.2975)	0.45008632	1.4403(0.5588~3.7123)
pT (ref = Tis)				
T1	0.99578437	581752.954(0~Inf)	0.99892672	7051868.7016(0~Inf)
T2	0.99577429	600514.5579(0~Inf)	0.99892434	7303453.8648(0~Inf)
T3	0.99550403	1406344.2441(0~Inf)	0.99886669	17036399.7058(0~Inf)
T4	0.99516369	4106845.2968(0~Inf)	0.99886077	18582803.6035(0~Inf)

Notes: The median expression of gene was used as the cut-off to divided patients into High or Low group.
CI = Confidence Interval.

Table S2. The univaraite and multivariate Cox regression model (TCGA_CESC n=110)

Variable	Univariate regression		Multivariate regression	
	P value	Hazard ratio (95%CI)	P value	Hazard ratio (95%CI)
FAM83A (High vs Low)	0.0019	1.61(1.21~2.14)	0.554146	0.8282(0.4436~1.5464)
Age (ref = <=46)				
>46	0.7464724	1.0971(0.6256~1.9238)	0.973298	1.0105(0.5467~1.8681)
Grade (ref = G1)				
G2	0.68099913	1.3561(0.3174~5.7937)	0.95323	1.0468(0.2268~4.832)
G3	0.30268294	2.1375(0.5041~9.0628)	0.484615	1.7233(0.3746~7.9288)
G4	0.99615114	0(0~Inf)	0.998339	0(0~Inf)
Unknown	0.92277067	0.9076(0.1278~6.4473)	0.235043	0.2808(0.0345~2.2846)
Stage (ref = I)				
II	0.28632993	0.6537(0.2993~1.428)	0.564171	0.6778(0.1807~2.5421)
III	0.15485529	0.4225(0.1289~1.3847)	0.015359	0.1629(0.0376~0.7066)
IV	0.00021401	4.4149(2.0112~9.691)	0.048914	5.6484(1.0083~31.6421)
Unknown	0.9956961	0(0~Inf)	0.997495	0(0~Inf)
pM (ref = M0)				
M1	0.41076343	1.8458(0.4285~7.9508)	0.286746	0.3075(0.0351~2.6923)
Unknown	0.61691348	1.1605(0.6477~2.0791)	0.786272	0.9068(0.447~1.8394)
pN (ref = N0)				
N1	0.01260335	2.4972(1.2168~5.1251)	0.010495	2.7329(1.2654~5.9023)
Unknown	0.06301106	1.88(0.9663~3.6574)	0.071365	2.5364(0.9222~6.9756)
pT (ref = Tis)				
T1	0.99692579	2344401.1893(0~Inf)	0.999044	4751343.9366(0~Inf)
T2	0.99699134	1714823.6429(0~Inf)	0.999056	3904707.0735(0~Inf)
T3	0.99691559	2461345.6419(0~Inf)	0.998983	12502764.7436(0~Inf)
T4	0.99656111	13356450.5688(0~Inf)	0.999023	6590499.7869(0~Inf)

Notes: The median expression of gene was used as the cut-off to divided patients into High or Low group. CI = Confidence Interval.
